# Molecular Simulation of Naphthalene, Phenanthrene, and Pyrene Adsorption on MCM-41

**DOI:** 10.3390/ijms20030665

**Published:** 2019-02-03

**Authors:** Xiong Yang, Chuanzhao Zhang, Lijun Jiang, Ziyi Li, Yingshu Liu, Haoyu Wang, Yi Xing, Ralph T. Yang

**Affiliations:** 1 School of Energy and Environmental Engineering, University of Science and Technology Beijing, Beijing 100083, China; yangx@ustb.edu.cn (X.Y.); frljiang@live.com (L.J.); ysliu@ustb.edu.cn (Y.L.); xing_bkd@163.com (Y.X.); 2 Beijing Higher Institution Engineering Research Center of Energy Conservation and Environmental Protection, Beijing 100083, China; 3 College of Biochemical Engineering, Beijing Union University, Beijing 100023, China; Chuanzhao.zhang@163.com (C.Z.); jdthaoyu@buu.edu.cn (H.W.); 4 Department of Chemical Engineering, University of Michigan, Ann Arbor, MI 48109, USA; yang@umich.edu

**Keywords:** polycyclic aromatic hydrocarbons (PAHs), molecular simulation, grand canonical Monte Carlo (GCMC), adsorption, mesoporous material

## Abstract

The adsorption of three typical polycyclic aromatic hydrocarbons (PAHs), naphthalene, phenanthrene, and pyrene with different ring numbers, on a common mesoporous material (MCM-41) was simulated based on a well-validated model. The adsorption equilibriums (isotherms), states (angle distributions and density profiles), and interactions (radial distribution functions) of three PAHs within the mesopores were studied in detail. The results show that the simulated isotherms agreed with previous experimental results. Each of the PAHs with flat molecules showed an adsorption configuration that was parallel to the surface of the pore, in the following order according to the degree of arrangement: pyrene (Pyr) > phenanthrene (Phe) > naphthalene (Nap). In terms of the interaction forces, there were no hydrogen bonds or other strong polar forces between the PAHs and MCM-41, and the O–H bond on the adsorbent surface had a unique angle in relation to the PAH molecular plane. The polarities of different H atoms on the PAHs were roughly the same, while those of the C atoms on the PAHs decreased from the molecular centers to the edges. The increasing area of the π-electron plane on the PAHs with the increasing ring number could lead to stronger adsorption interactions, and thus a shorter distance between the adsorbate and the adsorbent.

## 1. Introduction

Polycyclic aromatic hydrocarbons (PAHs) generally refer to hydrocarbons that have two or more benzene rings [1,2]. PAHs, commonly known as carcinogens, mutagens, teratogens, and bioaccumulators, can cause great damage in humans and the environment [3,4,5,6,7,8]. They are also a part of the atmospheric particulate pollutant PM_2.5_ [9]. In 1976, the United States Environmental Protection Agency (EPA)’s list of 129 “priority pollutants” included 16 types of PAH compounds [10]. Low-ring PAHs such as naphthalene (Nap), phenanthrene (Phe) and pyrene (Pyr) are the main components in the atmosphere [11]. The adsorption method has become one of the important ways to purify PAHs due to its high efficiency and low energy consumption [12,13,14]. Ordered mesoporous adsorbents have shown high adsorption capacities for their tailorable pore systems, especially in the adsorption of molecules such as PAHs [15,16]. Mastering the adsorption mechanism of PAHs on mesoporous materials is critical to optimize the material’s performance. Molecular simulation has obvious benefits in the adsorption isotherm calculation and adsorption state analysis of adsorption molecules. It can analyze the adsorption mechanism [17,18] and the nature of action in depth, and it has been widely used in adsorbent selection and design [19,20].

MCM-41 [21,22,23] is a typically ordered mesoporous adsorbent from a family of silicate and alumosilicate solids that has been employed to investigate the adsorption phenomena on various gases [24,25]. Since the emergence of MCM-41, a large number of adsorption-related material structures and physical and chemical properties have been analyzed [23,26]. Molecular simulations with MCM-41 have been conducted with the Monte Carlo (MC) method to calculate the various gas adsorption conditions [27,28,29,30,31]. The MCM-41 model evolved from earlier simple models [32,33,34,35,36] (e.g., single-layer cylindrical atomic wall, and the rough model of simple potential function), to later all-atom models [37,38] (e.g., carving out models in amorphous silica, and ensuring the bonding relationship between silicon and oxygen atoms), and recent templated synthesis models for restoring real synthetic conditions [39,40]. Among them, the carving-out modeling method is easier to implement, and calculations based on this model have been widely used for gas adsorption because of the good agreement with experimental results. Jing et al. [41] discussed the adsorption effects of two structural forms of unit cell models, and provided ideas for improving computational efficiency. Ugliengo et al. [42] optimized the MCM-41 structure by the density functional method. Pajzderska et al. [27] and Coasne et al. [43] studied the surface roughness, pore shape, and surface undulation of mesoporous channels, and made corresponding structural improvements. The above works have important significance in the improvement of the overall structure, surface morphology, and adsorption sites of the MCM-41 model.

With the development of molecular simulation technology, MCM-41 has made great progress in the field of adsorption separation as a typical mesoporous molecular sieve [28,29,44,45]. With regard to small molecule gas adsorption, Bonnaud et al. [46] characterized and analyzed the adsorption state and the diffusion phenomenon of water molecules in the pores of MCM-41 [46]. In the adsorption simulation of low-volatility compounds and macromolecular organics such as drugs, the adsorption characteristics of ibuprofen [47], tert-butanol [48], and neopentane [49] have been studied, as well as the influence of water molecules on adsorption [50,51]. Coasne et al. [30,31,52] studied the role of organic molecules such as benzene and toluene in the pores based on the combination of the angle of the benzene ring plane and the density profile. Compared with common small molecular gases, macromolecular organic compounds have more obvious molecular structures such as prominent geometric skeletons, polypolar sites, and deformations. It is beneficial to grasp the distribution of these properties in a spatial capacity to judge the state of the molecules in the pores and analyze the adsorption mechanism. At present, there are few simulation studies on the adsorption of PAHs. Analyses of the adsorption states and the adsorption mechanism are relatively lacking. Studying the adsorption of typical PAHs such as Nap, Phe, and Pyr on mesoporous adsorbents is helpful to understand the adsorption mechanism and its application in the removal of PAHs. Analysis of the state of molecules inside the adsorbent is extremely important for research and the improvement of adsorption efficiency.

In this paper, adsorption of the three PAHs Nap, Phe, and Pyr will be simulated based on the grand canonical Monte Carlo (GCMC) method. The potential adsorption mechanism will be discussed based on the adsorption states of the three molecules in the pores.

## 2. Results and Discussion

### 2.1. Adsorption Equlibrium

**Figure 1:**
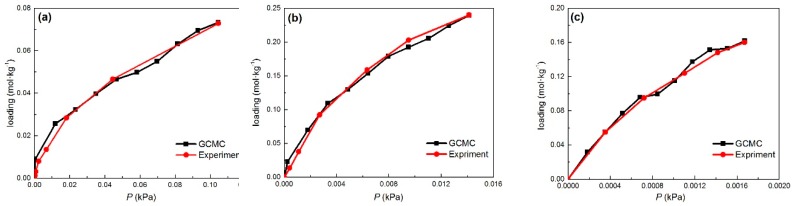
Adsorption isotherms for Nap, Phe, and Pyr on the MCM-41 model compared with experiment results at 398K. (**a**) Nap; (**b**) Phe; (**c**) Pyr; Key: red, grand canonical Monte Carlo (GCMC); black, experiment.

Figure 1 provides the GCMC simulation and experimental results [53,54] of the adsorption isotherms of Nap, Phe, and Pyr on MCM-41 at 398 K (normal flue gas temperature). The isotherms from the simulation calculation were in good agreement with the experimental values, with the following order according to the adsorption capacity: Pyr > Phe   > Nap, corresponding to the inverse sequence of the volatility of the PAH molecules. The results indicate that the current MCM-41 unit cell model with the calculation condition was reasonable and can truly reflect the adsorption of PAHs at low concentrations. The three isotherms of the PAHs belonged to Type I according to the IUPIC classification [55]. Because the PAH concentrations were low, all of the isotherms did not reach the saturated adsorption capacity. The adsorption capacity Q (mmmol·kg^−1^) can be determined with the formula:

(1) $$Q=1000*N_{PAH}/M_{adsorbent},$$

where $N_{PAH}$ is the number of PAHs in the pores of MCM-41 and $M_{adsorbent}$ (g·mol^−1^) is the molar mass of the adsorbent.

**Figure 2:**
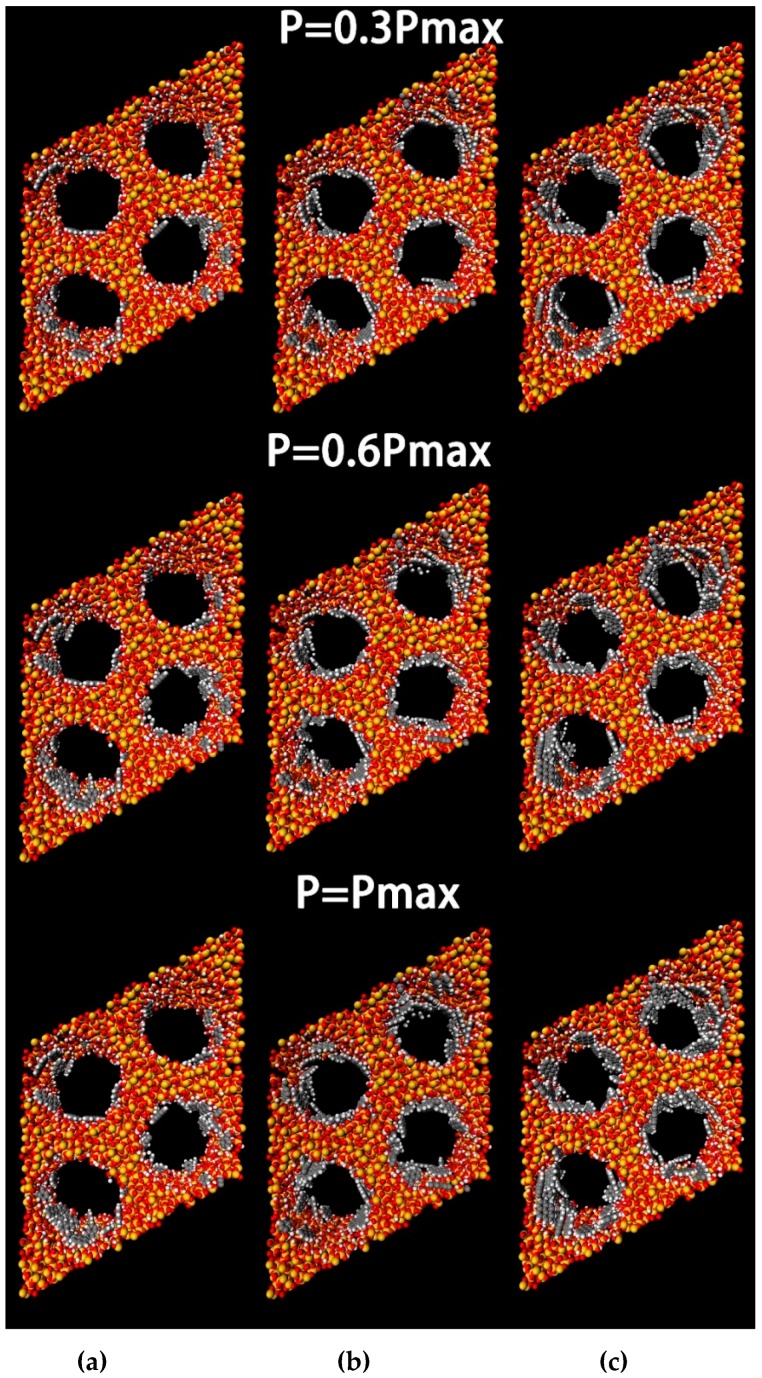
Typical configurations of Nap, Phe and Pyr adsorbed at 398K in the cylindrical nanopore of the model MCM-41. (**a**) Nap; (**b**) Phe; (**c**) Pyr, Key: Si, yellow; O, red; H, white; C, grey.

Figure 2 presents a diagram of PAH adsorption as an instantaneous map under different pressures, and it reflects the overall distribution state of PAH molecule adsorption in the cylindrical channel. PAH pore adsorption was mainly based on single-layer adsorption, and this also indicated that the isotherm belonged to Type I. It can also be observed that the PAHs were adsorbed on the surface of the mesoporous channel in a tiled manner. This will be discussed in the following section.

### 2.2. Adsorption State

#### 2.2.1. Angle Distribution

**Figure 3:**
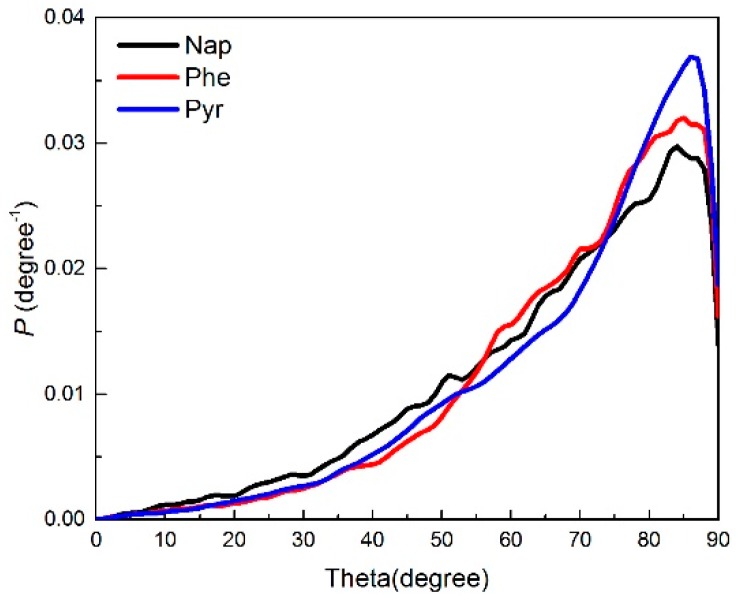
Angle distribution of Nap, Phe, and Pyr on the MCM-41 model. Key: black, Nap; red, Phe; blue, Pyr.

It was important to explore the angular distribution of PAH adsorption in the pores since the PAHs exhibited a planar molecular structure. Thus, the adsorption state and configuration of PAHs on the pore surfaces was statistically obtained. Here, the angle between the plane normal vector of the adsorbed PAHs and the axis direction of the cylindrical channel was defined as θ (theta). Figure 3 provides the angle distributions of Nap, Phe, and Pyr adsorption on MCM-41. The θ of the three PAH molecules as they adsorbed in the pore was mainly distributed between 80 and 90 degrees, and the probability of θ between 0 and 15 degrees was close to zero. This indicates that there are no PAHs perpendicular to the axial direction of the channel; that is to say, the PAHs are mainly adsorbed on the surface of the mesoporous channel in a tiled manner, which is consistent with Figure 3. The degree of the parallel distribution (θ tends to 90°) of adsorbate over the channel surface increased with increasing PAH weight, indicating a more stable interaction between the adsorbate and adsorbent.

In order to explain the orientation of the adsorbate molecules in the pores of MCM-41, an order index, S, was defined as follows [30]:

(2) $$S=\left(\frac{3}{2}\cos^{2}\theta -\frac{1}{2}\right)$$

where S tended to be −0.5 when the adsorbate molecules tended to be parallel to the surface, and S tended to be 1 when the adsorbate molecules tended to be perpendicular to the surface. The statistical results of the S values of the three PAHs are shown in Table 1. The S values of Phe and Pyr were close to −0.4, indicating that the two molecular planes were close to being parallel to the axial direction of the channel. The S value of Nap was only −0.2, indicating that the tiling property of the adsorption configuration on the surface was weak, and there was less molecular arrangement order compared to that of Phe and Pyr. The S values of the three PAHs did not reach the ideal value of −0.5, probably because the thermal motion of the molecule at a higher temperature of 398 K caused local perturbations of the molecule at the adsorbed position. In this study, the adsorbates were at low gas concentrations, and monolayer adsorption dominated. Three non-polar PAH molecules exhibited relatively weak physical adsorption properties on the surface. Therefore, the angle and position of their distribution may also be disturbed by the interaction of the surrounding adsorbate molecules.

#### 2.2.2. Density Profile

**Figure 4:**
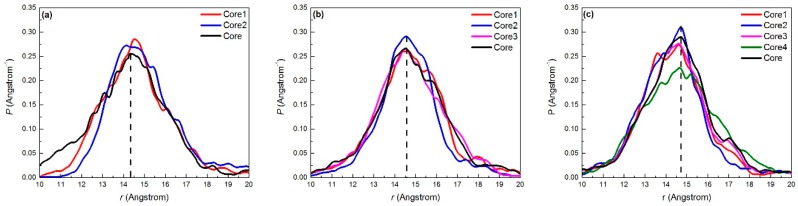
Density profiles for diffident centers in (**a**) Nap; (**b**) Phe; (**c**) Pyr at 398K on the MCM-41 model.

**Figure 5:**
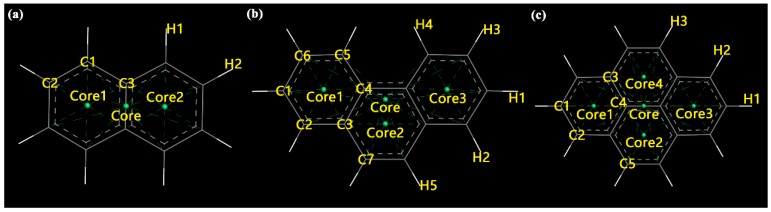
Graphical site description of polycyclic aromatic hydrocarbon (PAH) molecules. (**a**) naphthalene; (**b**) phenanthrene; (**c**) pyrene; Key: CoreX, the benzene ring center (X is the number); yellow, symbol of different atoms.

The density profile can be used to infer the average spatial position of the molecules in the pores by overlapping the profiles of the single-peak type, symmetric double-peak type, and irregular type, which can be used to further analyze the adsorption. Figure 4 shows plots of the central density profile of the three PAHs adsorbed on MCM-41. The abscissa indicated the radial direction of the mesoporous cylindrical channel, and the origin represented the center of the channel. The maximum distance was almost equal to the radius of the pores in the MCM-41 model (around 19 Å) in our simulation; the further away the molecule was from the center of the pores, the closer it was to the edge of the pores. For the special structure of the three PAHs (two or more benzene ring structures), the density distributions of different geometric centers on Nap, Phe, and Pyr, as shown in Figure 5, were refined to further gauge the state of adsorbed molecules. 

The molecules of Nap, Phe, and Pyr are stable planar molecules, and there are three special states in the adsorption process [49], which are in parallel with the molecular plane, perpendicular to the surface of the molecular sieve, and exist at a unique angle to the surface. These three different states correspond to three different interactions: the weak interaction between the surface of the adsorbent and the molecular plane, the strong interaction between the surface of the adsorbent and the polar edges of the molecules, and the combination of the two effects.

Figure 4 shows that the density distributions of all centers did not exhibit obvious symmetrical bimodal phenomena. The larger the molecular radius, the farther the distance of the density distribution peak corresponding to the center of the pore (the corresponding distances from the center of benzene rings to the central axis of the pore were 14.35 Å, 14.65 Å, and 14.75 Å), and the stronger the interaction between the molecule and the surface. The peak position relationship of the density profile between the adsorbate molecules Core and CoreX illustrates the following three points. First, with increasing adsorbate molecular size, there was a greater coincidence that the peak position of the density was located between the centers of the benzene ring and the center of the molecule. This corresponded to an increase in the stability of the molecular plane with the edge of the PAHs likely to be deflected by the surface polarity interaction. Second, the two sub-centers of the Nap molecule were partially offset from the peak position of the density at the center, indicating that there was a significant tilt angle between the Nap molecule and the surface of the pore. Third, the difference between the density peak positions of the centers of the benzene ring and the center of the Phe and Pyr molecules was small. It can be concluded from the statistical results of the previous angle distributions that the parallelism between the molecular planes and the surface of the pore was satisfactory.

### 2.3. Adsorption Interactions

**Figure 6:**
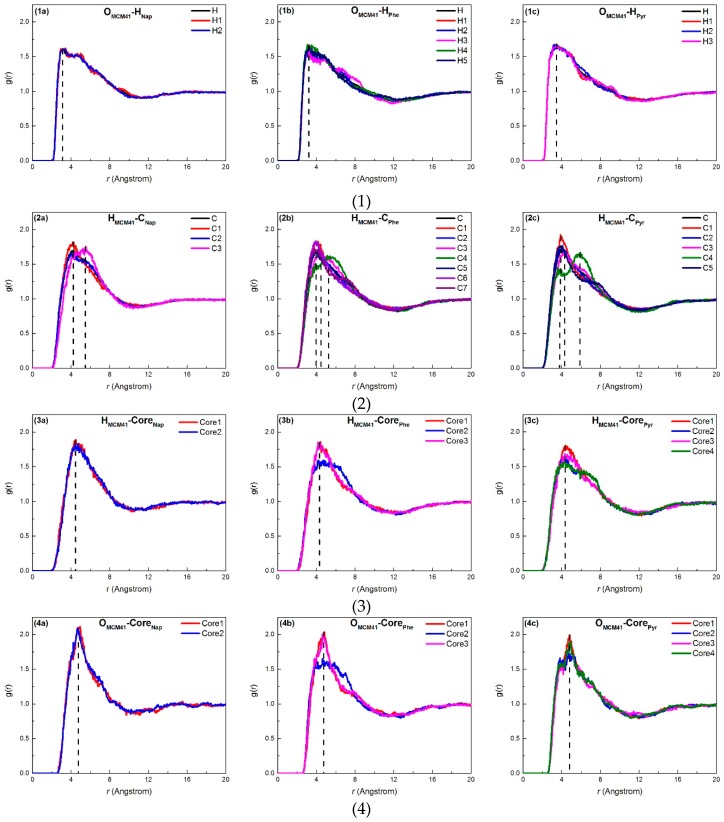
Radius distribution function for (**a**) Nap, (**b**) Phe, and (**c**) Pyr. (**1**) O atoms on MCM-41 and H atoms on PAHs, (**2**) H atoms on MCM-41 and C atoms on PAHs, (**3**) H atoms on MCM-41 and centers on PAHs, and (**4**) O atoms on MCM-41 and centers on PAHs.

The radial distribution function (RDF, g(r)) is a function of the distance between different particles (molecules or atoms). It is the ratio of the local density to the average density, which indicates the density variation of the particle in space. The RDF method was employed to further analyze the distribution distance between the adsorption sites of the PAHs (rings or atoms) and the active atoms on the adsorbent surface, such as O and H. The RDF between the PAH molecules and different sites of the mesoporous surface is shown in Figure 6. The distances corresponding to the RDF peaks are summarized in Table 2.

Figure 6(1) shows that the H atoms on the PAHs at different positions were the same distance from the O atoms on the surface of MCM-41. This indicated that the H atoms in the same PAH molecule had similar weak activities. The peaks of the RDF distances corresponding to Nap, Phe, and Pyr were 3.43 Å, 3.63 Å, and 3.53 Å, respectively (see Table 2), which were all larger than the common hydrogen bond length (≤3.10 Å). This indicated that there could be limited hydrogen bonds produced between PAH molecules and surface silanol groups (Si–OH).

Figure 6(2) shows there were large differences in the distances between the H atoms on MCM-41 and C atoms (H_MCM41_–C_orePAHs_) at different positions on the PAHs. The C atoms of the C–H bonds on the PAH were close to H atoms on MCM-41, and C atoms near the center of the PAH were spaced apart from the H atoms on MCM-41. This distribution and action was a result of the positive and negative charges of the molecule. The H and O atoms on the PAHs or the surface of MCM-41 had positive potential and negative potential electrical properties, respectively, while the electrical property of C atoms on the PAHs was dependent on the positions. The central C atom was positively charged while the outer C atom was negatively charged.

Table 2 shows that the difference in the distance between H and O atoms on MCM-41 and the RDF peak of the PAH centers was 0.44 Å (Nap), 0.4 Å (Phe), and 0.45 Å (Pyr). They were smaller than the average distance between H and O atoms in the surface silanol group (0.94 Å), indicating that the molecular planes of the O–H bond and the PAHs were not perpendicular, but existed at a unique angle. The spacing between the central PAH ring and the H and O atoms of the PAHs decreased with increasing PAH molecular size. The C atoms near the center of the PAHs, which were negatively charged, attracted Si and H atoms that were positively charged, and this led to a decrease in the distance between the PAHs and the inner surface of the adsorbent. This result was consistent with the density peak position results shown in Figure 4.

**Figure 7:**
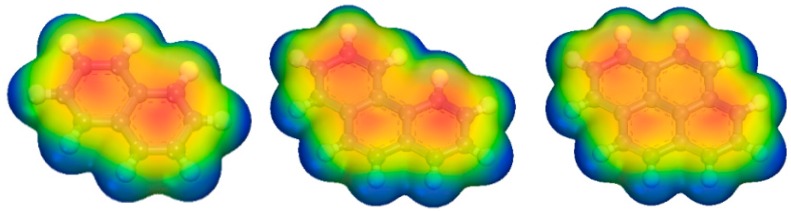
Electrostatic potential and charge density distribution results of Nap, Phe and Pyr. The electrostatic potential distribution ranged from −0.03 to 0.03, with the blue portion representing the positive potential and the red portion representing the negative potential.

It can be concluded from the angular distribution and density peak position analysis that the three PAH molecules were adsorbed in a flat position on the inner surface of the adsorbent tunnel. The adsorbates possessed a stable planar structure with H atoms on the molecular edge, and there was no strong polar force generated during the adsorption process that can result in the severe tilt or deflection of the molecular plane. These findings were proved by RDF results, where the hydrogen bonding was weak at the edge of the PAHs from the large distance between H atoms on PAHs and O atoms on MCM-41. Additionally, there was no unique H atom, and the surface generated a prominent force that tilted the PAH plane for the same activity of H atoms at the edge of the PAHs. As we know, the current PAHs were formed by the combination of benzene rings, and large π bonds easily form throughout the molecule. The potential and charge density distributions of Nap, Phe, and Pyr obtained by the DFT simulations, shown in Figure 7, illustrated that the center of the ring on both sides of the PAH molecular plane was negatively charged (red part in Figure 7), which is a neighboring π bond, and the area of action of the π-electron plane increased when the number of rings increased. This led to enhanced, or stronger, adsorption when the adsorption process was governed by dispersion forces [56]. Hence, the heavier the molecular weight of the PAHs, the stronger the interaction with the surface of the pore and the shorter the distance between the adsorbate and adsorbent.

## 3. Methods and Parameters

### 3.1. Model Structures

**Figure 8:**
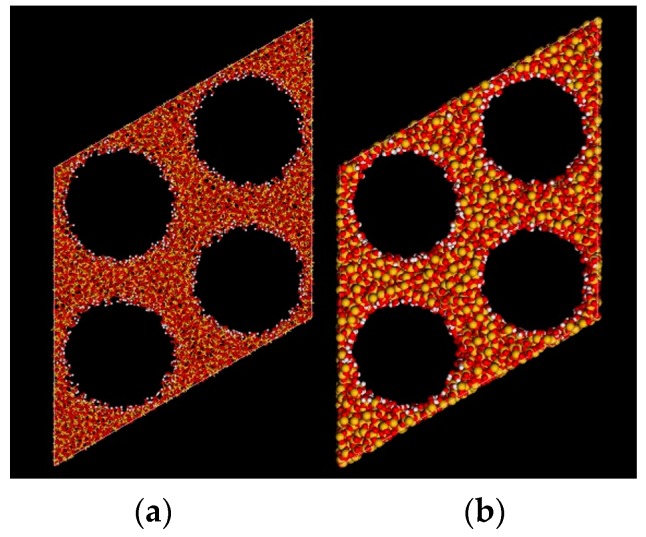
The final structure of the MCM-41 model. (**a**) Ball-and-stick model; (**b**) CPK model; silicon (Si), yellow; oxygen (O), red; hydrogen (H), white.

An all-atom model of MCM-41 was established with the carving-out method [36,37] based on the rhombohedral cell model [57], as shown in Figure 8. The main mesoporous structure of the model consisted of excavated cylindrical holes in amorphous silica. Each unit contained four cylindrical channels. The atomic bonds in the whole unit cell were saturated by the connection of the oxygen (O) and hydrogen (H) atoms to the unsaturated bonds from silicon (Si) and O atoms (the Si atoms with three OH atoms were removed). The model was optimized and relaxed based on interactions described by the Dreiding force field. The ESP charges were calculated according to the method from Zhuo et al. [44] to finalize the structure of MCM-41. The model structure parameters are shown in Table S1, and comparisons of the characterization results with experimental values [52] are shown in Figure S1 in the Supplementary Material.

**Figure 9:**
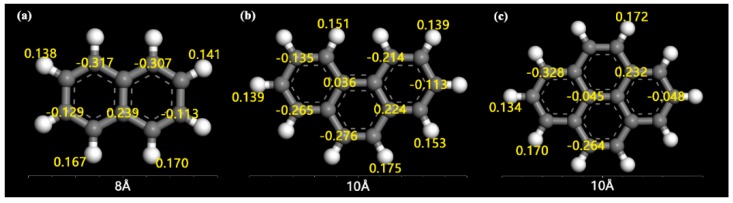
The structures of naphthalene (Nap), phenanthrene (Phe), and pyrene (Pyr) with ESP charges. (**a**) Nap; (**b**) Phe; (**c**) Pyr; Key: carbon (C), grey; H, white; charges, yellow.

Nap, Phe, and Pyr are typical benzene-ring structures, as shown in Figure 9. The molecular models of the three PAHs were determined with the density function theory (DFT) method, with all-electron atomic charges obtained by the ESP method. In the DFT calculation, the Perdew–Wang method was employed to exchange correlation functions in the calculation, where the base group used was double numerical plus polarization (DNP). The distribution of the electrostatic potentials on the electron isopycnic surface of the three PAHs is shown in Figure 7.

### 3.2. Calculation Method

In the GCMC simulation, the adsorption temperature was fixed at 398 K, and the adsorbate molecules were regarded as rigid molecules. The Dreiding force field was used to describe the energy forms of the adsorbent–adsorbate and adsorbate–adsorbate interactions. The atomic force field form and interaction parameters are shown in Table S2. Electrostatic interactions are described in Ewald format with an accuracy of 10^−4^ kcal mol^−1^. The cutoff radii for the van der Waals interactions and hydrogen bonds were 15.5 Å and 4.5 Å, respectively. The total step number was 1 × 10^7^, in which the first 5 × 10^6^ steps were used to balance the adsorption state, and the next 5 × 10^6^ steps were used to calculate the state average of the entire ensemble. The PAHs’ gas partial pressure in the simulation was set at a lower level (~100 ppm) according to the common concentration ranges of PAHs in actual situations [58]. The fugacity value required in the calculation process was obtained by converting the pressure into the physical parameters of Nap, Phe, and Pyr by the Peng–Robinson equation. The parameters such as critical temperature, critical pressure, and the eccentricity factor are shown in Table S3.

The adsorption model results under the pressure of 0.8 P_max_ were used to further analyze the adsorption state of the three PAH molecules in the main channel of MCM-41. The adsorption states including the angle distribution and the density peak were analyzed. The radius radial distribution function representative of adsorbate–adsorbent mutual force was finally discussed.

## 4. Conclusions

Based on the GCMC simulation, the MCM-41 model was established, and the adsorption of Nap, Phe, and Pyr on MCM-41 was simulated. The adsorption model was validated by use of experimental adsorption isotherms. During the adsorption, no perpendicular state between the PAH molecular plane and the central axis of the adsorbent channel was observed. The order according to the degree of adsorption was: Pyr > Phe > Nap. The three PAHs were adsorbed in a flat position on the surface of the pore, and the configuration planes of Phe and Pyr were more parallel to the surface compared to Nap. The RDF curves indicated that there was no hydrogen bonding produced during the adsorption process. The H atoms in the same PAHs had the same activity, but the adsorption activity of the C atoms decreased from the central position, and the O–H bond on the adsorbent surface had a unique angle with the plane of the PAHs. The area of action of the π-electron plane increased when the number of rings increased, leading to enhanced, stronger adsorption, and thus, a shorter distance between the adsorbate and adsorbent.

## Figures and Tables

**Table 1:** Order parameter (S) for the molecular direction of Nap, Phe, and Pyr.

Adsorbate	S	S Values Corresponding States	
Nap	−0.1843	0	disordered
Phe	−0.3568	1	vertical
Pyr	−0.3629	−0.5	parallel

**Table 2:** Distances between the H or O atom of MCM41 model and CoreX of the three PAHs.

Type	Distance (Å)		
	Nap	Phe	Pyr
O_MCM41_–H_PAHs_	3.43	3.63	3.53
H_MCM41_–Core_PAHs_	4.48	4.43	4.38
O_MCM41_–Core_PAHs_	4.92	4.83	4.83
O_MCM41_–H_MCM41_	0.94		

## Supplementary Materials

Supplementary materials can be found at http://www.mdpi.com/1422-0067/20/3/665/s1.

## Abbreviations

Nap	Naphthalene
Phe	Phenanthrene
Pyr	Pyrene
GCMC	Grand Canonical Monte Carlo
