# Supplementary material for: Molecular Simulation of Naphthalene, Phenanthrene, and Pyrene Adsorption on MCM-41
Source: Int J Mol Sci. 2019 Feb 3;20(3):665. doi: 10.3390/ijms20030665 (PMC6387010; doi:10.3390/ijms20030665)
Supplement: Supplementary file 1 [file ijms-20-00665-s001.pdf]

International Journal of *Molecular Sciences*

MDPI

## Supplementary materials

**Table 1.** Structure properties of the MCM-41 model.

| Properties | MCM-41 |
|---|---|
| Cell parameters(Å) | (89.021, 89.021, 37.057) |
| Lattice angle(°) | (90, 90, 120) |
| Cell volume(Å$^3$) | 254325 |
| Number of O atoms only connecting Si | 5612 |
| Number of O atoms connecting H | 1042 |
| Number of Si atoms | 3070 |
| No. of H atoms | 1042 |
| Wall thickness(Å) | 11.51 |
| T sites(/1000Å$^3$) | 7.5 |
| Skeleton density(g/cm$^3$) | 2.2134 |
| Porosity density(g/cm$^3$) | 1.2648 |
| Pore radius(Å) | 33 |
| Connolly surface(Å$^2$) | 22459.73 |
| Surface Area(m$^2$/g) | 698.269 |
| -OH groups(/nm$^2$) | 4.64 |

**Table 2.** Lennard-Jones potential parameters and atomic charges.

| Molecule | Site | σ(Å) | ε/kB(K) | *q*(e) |
|---|---|---|---|---|
| | Si | 3.804 | 155.858 | 1.24152 |
| MCM-41 | O | 3.033 | 48.115 | -0.73843 |
| | H | 2.846 | 0.0503 | 0.41006 |
| naphthalene | C | 3.473 | 47.813 | See figure 2 |
| phenanthrene pyrene | H | 2.846 | 0.05 | |

**Table 3.** Properties of naphthalene, phenanthrene and pyrene.

| Properties | Naphthalene | Phenanthrene | Pyrene |
|---|---|---|---|
| Molecular formula | $C_8H_{10}$ | $C_{14}H_{10}$ | $C_{16}H_{10}$ |
| Molar mass(g/mol) | 128.174 | 178.233 | 202.255 |
| Standard boiling point | 217.989 | 340.3 | 394.8 |
| Critical temperature(K) | 475.25 | 596.1 | 662.85 |
| Critical pressure(MPa) | 0.0698 | 0.0723 | 0.093 |
| Acentric factor(kJ/(kg·K)) | 0.302 | 0.499 | 0.5088 |
| Dipole moment(D) | 0 | 0 | 0 |

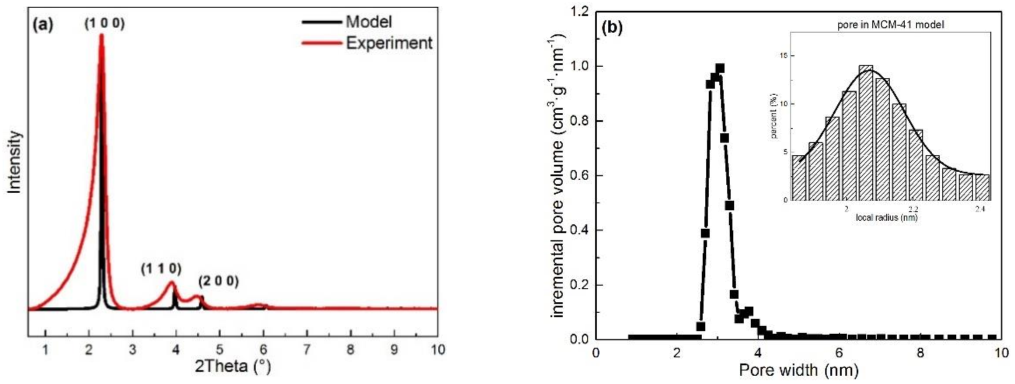


**Figure 1.** Characterization results of the MCM-41 model compared with experiments: (a) X-ray diffraction, (b) pore size distribution.
